# Structure and Selected Properties of Coatings Deposited by Arc Spraying Under in Inert Atmosphere Containing In Situ Fabricated Fe-Al Intermetallic Phases

**DOI:** 10.3390/ma18030646

**Published:** 2025-01-31

**Authors:** Paweł Kołodziejczak, Mariusz Bober, Tomasz M. Chmielewski, Michał Baranowski

**Affiliations:** Faculty of Mechanical and Industrial Engineering, Warsaw University of Technology, Narbutta 85, 02-524 Warsaw, Poland; pawel.kolodziejczak@pw.edu.pl (P.K.); tomasz.chmielewski@pw.edu.pl (T.M.C.)

**Keywords:** thermal spraying, iron aluminide, intermetallic phases, protective coatings, surface modification

## Abstract

Intermetallic compounds from the Fe-Al system are attracting increasing attention due to their outstanding properties, including excellent mechanical performance, low density, corrosion, and oxidation resistance, as well as resistance to sulfidation, carburization, and wear at elevated temperatures. These unique characteristics make Fe-Al intermetallics promising candidates for high-temperature and harsh environmental applications. However, challenges such as brittleness and low plasticity have hindered their broader use. By exploring the impact of spray conditions on coating properties, this study contributes to enhancing the performance and functionality of Fe-Al coatings in industrial applications, where durability and resistance to extreme conditions are essential. This article presents the results of research on the production of composite coatings from the Fe-Al system with in situ fabricated intermetallic phases. For this purpose, arc spraying in an inert gas was used. The coating manufacturing process was carried out by simultaneously melting two different electrode filler wires, aluminum and steel, in a stream of argon. The obtained coatings were subjected to tests of roughness, adhesion to the substrate, and microstructure. It was shown that both the roughness and adhesion to the substrate of coatings sprayed in air are higher than those sprayed in argon. The increase in roughness results from the greater oxidation of coatings sprayed in air, while better adhesion is the result of the formation of coatings at a higher temperature. Metallographic studies have shown that during the spraying process, the in situ synthesis of intermetallic phases occurred. The results showed the local occurrence of intermetallic phases from the Fe-Al system. Among the two dominant phases, i.e., Al and the Fe alloy, there are also the following phases: FeAl_3_, FeAl_2_, and Fe_2_Al_5_. Furthermore, in layers sprayed in an inert atmosphere, the share of oxides is small.

## 1. Introduction

Iron aluminide intermetallic compounds are generating growing interest due to their unique properties, including excellent mechanical properties, low density, outstanding resistance to corrosion and oxidation, resistance to sulfidation and carburization, and wear resistance even at elevated temperatures and low cost [1,2,3]. Due to these attractive properties, intermetallic phases from the Fe-Al system can replace stainless steels operating at elevated temperatures and, in particular, in harmful environments [3]. Moreover, previous studies have shown that the brittleness and low plasticity of these intermetallic phases can be significantly improved by the addition of Cr [4], heat treatment [5], grain refinement, or oxide dispersion strengthening [6]. Since the surface layer of machine parts is particularly exposed to mechanical loads, wear, and aggressive environments, FeAl phases are often used as protective coatings on critical parts and devices [4]. Coatings with a Fe-Al intermetallic phase structure are applied to components such as power boiler elements, hot sections of gas turbine engines, hot-gas filters, heat exchangers, recuperators, and combustor components in engines [2,4,7].

Intermetallic coatings are produced using various methods, mainly thermo-mechanical methods [8] and those related to welding processes [9,10]. The laser cladding of intermetallic powder coatings is commonly used for this purpose [11,12]. The deposition of intermetallics is most often carried out using various types of thermal spraying [13,14]. The HVOF spraying method is one of the most frequently used for intermetallics deposition [15,16], followed by cold spray [17,18,19] and, less frequently, plasma spraying [1,20] and D-gun [21,22]. All of the mentioned deposition methods involve spraying powders with an intermetallic structure. Intermetallic powders are a relatively expensive coating material, although they have a precisely defined phase structure and microstructure.

Studies on the production of intermetallic phases in situ by the arc method are known from the literature [23]. Shen et al. [24] obtained iron aluminide in the additive manufacturing process by simultaneously melting two wires of Al and Fe using the TIG method. In turn, by melting wires of Fe and Ni in an electric arc, intermetallic phases of the Fe-Ni system were produced [25]. In this way, phases of the Fe3Al and Fe-Ni systems were obtained, respectively, at a lower cost compared to traditional powder metallurgy processing. The arc spraying method is rarely used due to the limited possibility of applying coating material in the form of ready-made Fe-Al intermetallics. However, in the work [26], intermetallic phases were produced using this method through the simultaneous melting of two different wires of Fe and Al. Fe_x_Al_y_ phases are created during spraying or after spraying during additional heat treatment [27]. An obstacle to the synthesis of intermetallic phases in the coating may be the oxide phase formed during spraying in an air atmosphere [24]. Therefore, the main purpose of this article is to perform the arc spraying of a Fe-Al coating in a chemically inert gas environment of argon and to describe the impact of the argon shield on the properties of the coating compared to a coating sprayed in an air atmosphere.

## 2. Materials and Methods

**Figure 1:**
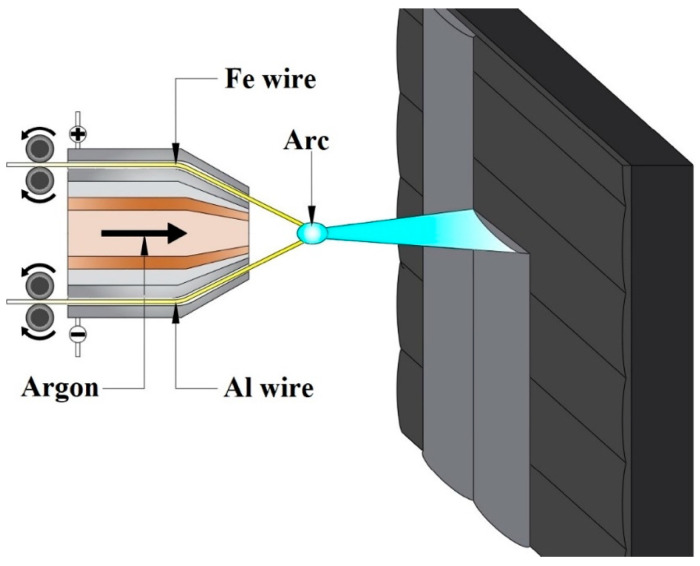
Scheme of the arc spraying process with Al and Fe wires.

The arc spraying process was performed using an MCP/TAFA 8830 model (ESAB Welding & Cutting Products, Florence, SC, USA) device. Two different electrode wires were used for spraying: an aluminum wire and a steel wire with a diameter of 1.6 mm. The chemical composition of these wires is provided in Table 1. During the spraying process, the ends of these wires were simultaneously melted in an electric arc (Figure 1). The substrate material consisted of steel plates commonly used for the production of energy infrastructure, designated 16Mo3 (according to [28]). The chemical composition of this steel is provided in Table 2. Samples with dimensions of 50 mm × 100 mm × 4 mm were prepared for the tests. Before the spraying process, the surface of the steel samples was subjected to abrasive blasting to remove impurities and prepare the surface. The spraying process was begun by heating the substrate to a temperature of 80 °C. Then, the first part of the coating was sprayed onto the surface of the sample in two passes: a horizontal pass followed by a vertical pass. Care was taken to ensure that the temperature did not exceed 250 °C. For sample 1Ar, a single-stage spraying process was completed. For sample 2Ar, a two-stage process was applied, with two additional spraying passes (one vertical and one horizontal), while for samples 3Ar and 3Air, the process consisted of three stages, resulting in four additional passes (two vertical and two horizontal). Between each spraying stage, the substrate was cooled down to a temperature of 80 °C. Surface temperature control was carried out using a pyrometer. The parameters of the arc spraying process are provided in Table 3. Coatings were made using argon (1Ar, 2Ar, 3Ar) or compressed air (3Air) as the gas transporting the molten metal.

After the spraying process, the obtained FeAl coatings were subjected to roughness tests using the Sensofar S Neox 3D Optical Profiler with the SensoSCAN software (1.9.2.0), utilizing active illumination focus variation technology. It is based on Sensofar’s extensive expertise in the field of combined confocal, focus variance, and interferometric 3D measurements. The uncertainty of the used measurement system (U), according to [29], for the used step height equals 0.07 µm. The roughness measurements for each sample were taken at three different locations under the following conditions: topography—1354 × 1018 pixels; area—1.75 mm × 1.31 mm; pixel size—1.29 μm/pixel; and magnification—10.00×.

**Figure 2:**
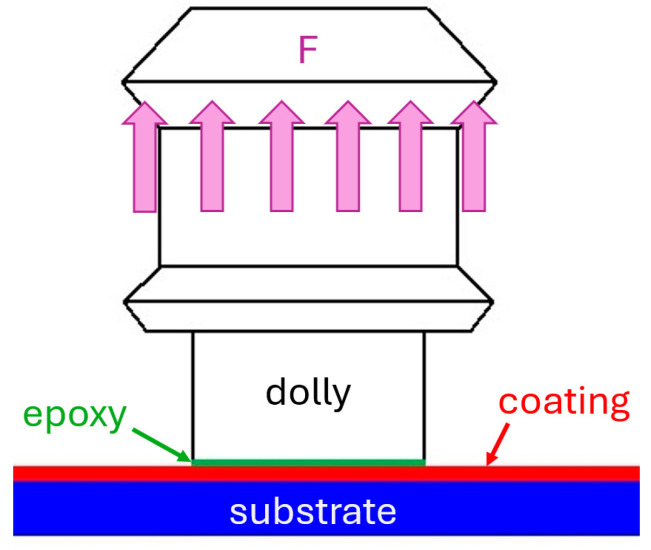
Thermally sprayed coating adhesion test diagram.

An Elcometer 510 automatic pull-off adhesion gauge (Elcometer, Manchester, UK)was used to measure the adhesive strength of the Fe-Al coatings on the steel substrate. During the tests, dollies with a diameter of 10 mm were used, providing a measuring range of 8 to 100 MPa. The device employs the pull-off method, in which the dollies were glued to the unprocessed sprayed coating with epoxy-based adhesive. The diagram of the test with the designation of the individual layers of the sample is shown in Figure 2. The pull rate was applied at 0.8 MPa/s. After the adhesion tests, to determine the nature of the failure, observations were made using an Olympus SZ61 stereoscopic microscope (Olympus, Hamburg, Germany).

Microstructure studies of arc-sprayed Fe-Al coatings were carried out on etched cross-sections. These samples were prepared according to a standard procedure. The studies were conducted using both optical and scanning microscopy. The microstructure was observed using an Olympus BX51 (Olympus, Hamburg, Germany) microscope and an Axia ChemiSEM (Thermo Fisher Scientific, Hillsboro, OR, USA) equipped with an EDS detector. The phase composition of the tested samples was determined using a Bruker D8 Advanced X-ray diffractometer (Bruker, Billerica, MA, USA). Furthermore, the thickness, porosity of the coatings, and volume fraction of intermetallic phase compounds were determined from the cross-section using Olympus Stream Essentials software (2.5.2).

## 3. Results and Discussion

### 3.1. Microstructure Investigations

**Figure 3:**
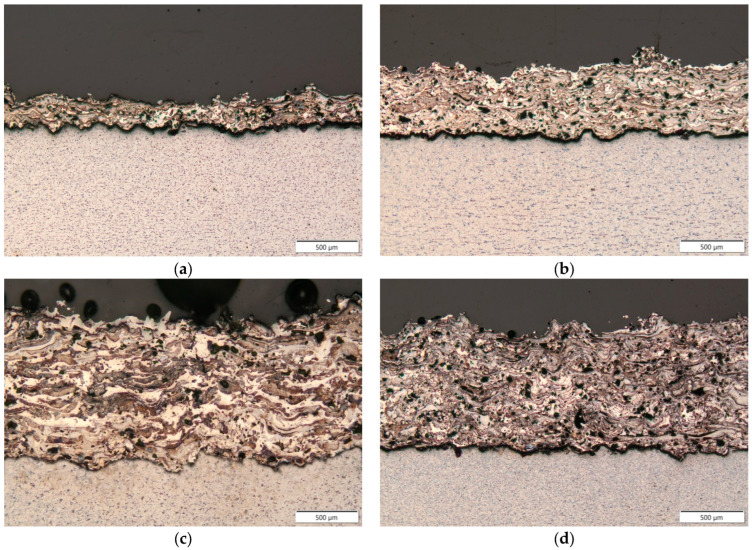
Macrostructures of Fe-Al sprayed coatings: (**a**) 1Ar, (**b**) 2Ar, (**c**) 3Ar, and (**d**) 3Air.

Figure 3 shows the macrostructures of arc-sprayed Fe-Al coatings using Ar (Figure 3a–c) and air (Figure 3d) as the spraying gasses. Moreover, the samples sprayed in argon were applied as single-stage (Figure 3a), double-stage (Figure 3b), and three-stage (Figure 3c) coatings. The macrostructures presented in Figure 3 exhibit variable layer thicknesses. The results of these tests are presented in Table 4, which shows average values from five measurements. The porosity of each coating was determined from five areas covering the entire thickness of the coating, and the average of these measurements was then calculated (Table 4). In the Fe-Al coatings sprayed in argon, the porosity remained at a similar level, approximately 10%. This indicates consistent conditions during the spraying process. In contrast, in the coating sprayed with compressed air, the porosity value was slightly higher, fluctuating around 12%.

**Figure 4:**
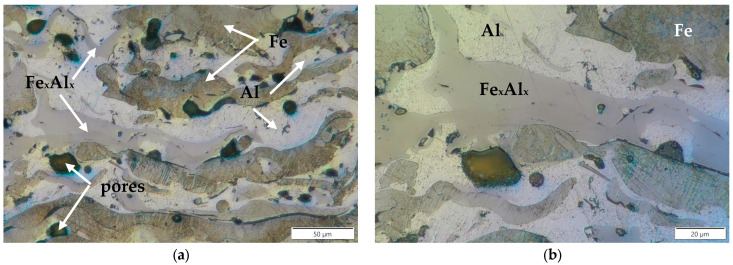
Banded microstructure of Fe-Al coatings (**a**) and the formation of new phases based on Fe and Al (black arrows indicate Fe_x_Al_x_ intermetallic phases) (**b**).

**Figure 5:**
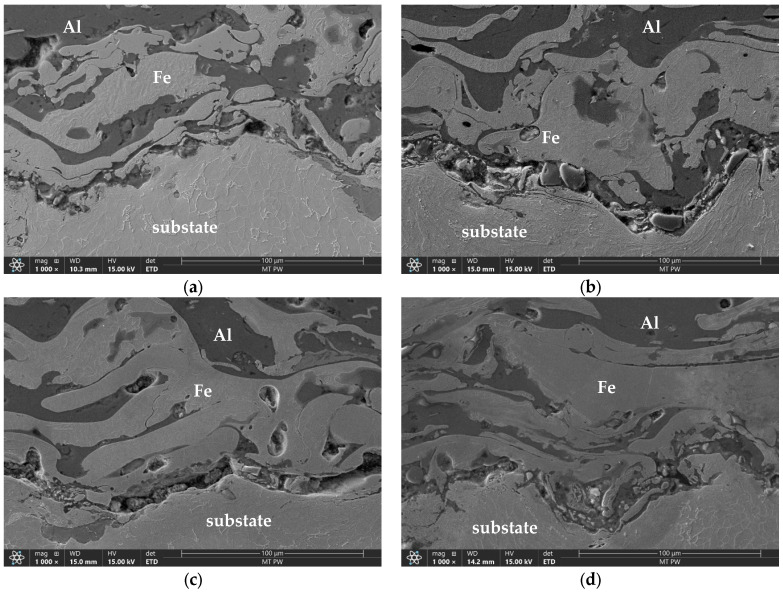
Coating–steel substrate interface sprayed in argon, namely (**a**) 1Ar, (**b**) 2Ar, and (**c**) 3Ar, and in air, (**d**) 3Air.

Observations carried out at higher magnification revealed the microstructure of the coatings (Figure 4). This structure is layered, with a clear dominance of two phases: dark and light. These phases are evenly distributed (Figure 4a). Pores (black areas), both small and large, spherical and elongated, are also visible. Moreover, against the background of the light phase, slightly darker elongated precipitates of the newly formed phase are visible (Figure 4b). It can be assumed that these are solid solutions of Al in Fe or intermetallic phases formed in situ from these elements. More detailed microstructure studies were conducted using scanning electron microscopy. Figure 5 shows the coating–substrate interface. In each of the tested samples, this boundary is continuous. However, a more refined microstructure was observed in the layer at the boundary with the steel substrate than in the interior of the coating. Occasionally, the small phases located at the substrate-coating interface had shapes close to spherical. This suggests the rapid crystallization of the coating material upon contact with the substrate material. The fast crystallization is most likely caused by the expanding argon. As a result, the falling particles of the filler material crystallize faster and do not flatten out. For the same reason, pores form near the boundary with the substrate (Figure 5a–c). In contrast, in the coatings sprayed with compressed air, although a fine structure was also observed near the boundary with the substrate, the melted particles are flattened and well-adapted to the steel surface, resulting in the absence of pores (Figure 5d).

**Figure 6:**
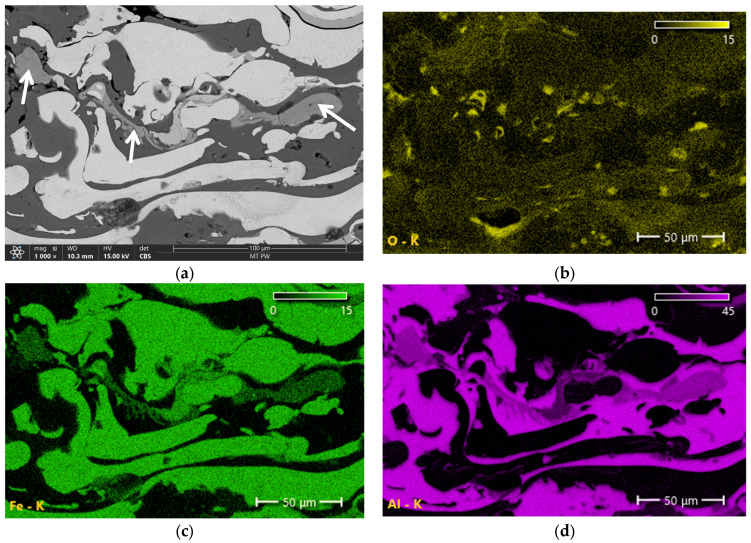
Microstructure of the 1Ar sample (**a**) and surface element distributions: (**b**) O, (**c**) Fe and (**d**) Al. The arrows indicate Fe_x_Al_x_ intermetallic phases.

**Figure 7:**
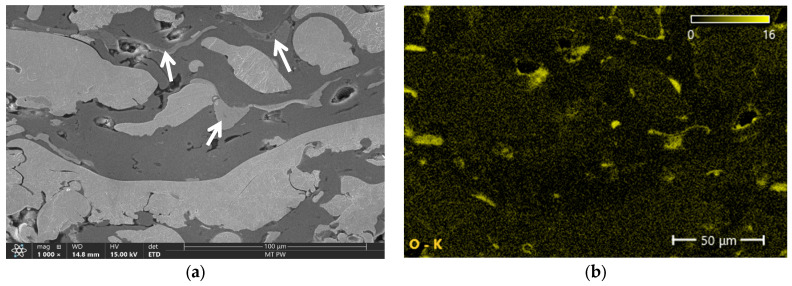

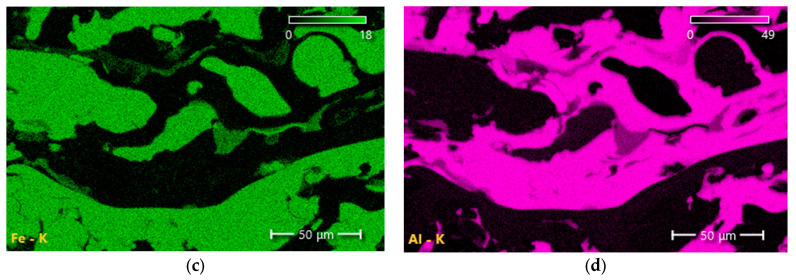
Microstructure of the 2Ar sample (**a**) and surface element distributions: (**b**) O, (**c**) Fe and (**d**) Al. The arrows indicate Fe_x_Al_x_ intermetallic phases.

**Figure 8:**
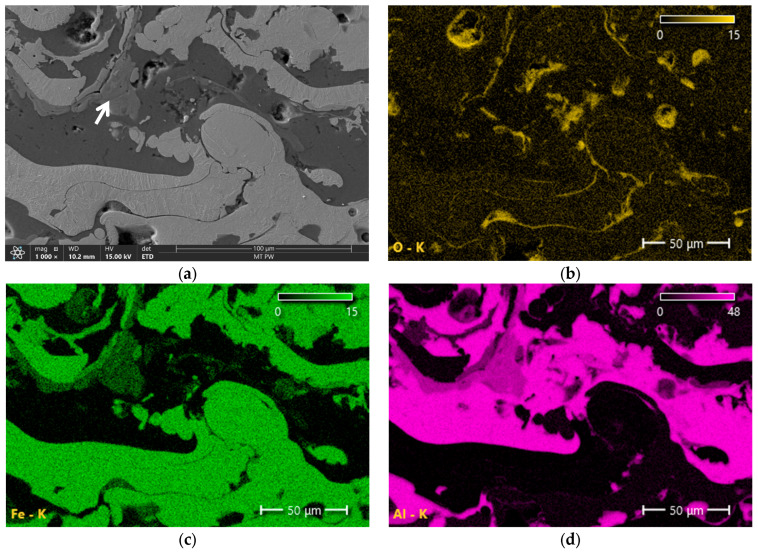
Microstructure of the 3Ar sample (**a**) and surface element distributions: (**b**) O, (**c**) Fe and (**d**) Al. The arrow indicates Fe_x_Al_x_ intermetallic phase.

**Figure 9:**
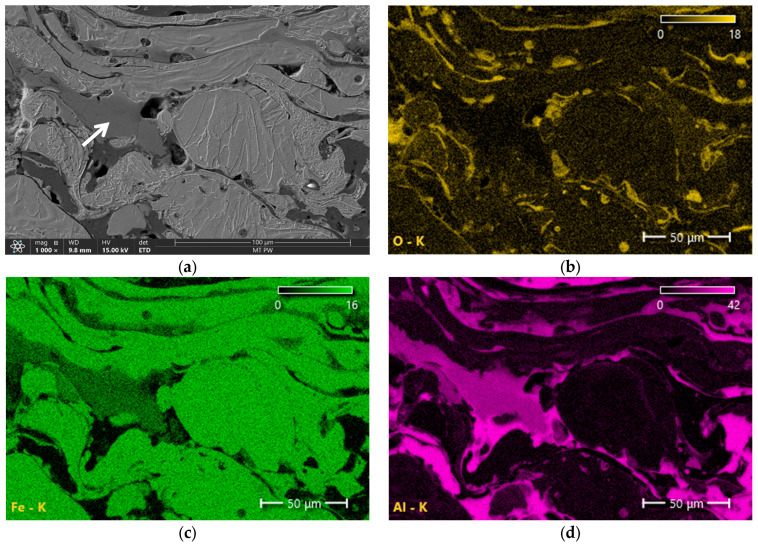
Microstructure of the 3Air sample (**a**) and surface element distributions: (**b**) O, (**c**) Fe and (**d**) Al. The arrow indicates Fe_x_Al_x_ intermetallic phase.

Figure 6, Figure 7, Figure 8 and Figure 9 show the microstructures of Fe-Al coatings from the zone located in the middle of the layer thickness and the surface distributions of oxygen, iron, and aluminium. The microstructures are clearly dominated by two phases, light and dark. The surface distribution of elements shows that the light phase is iron and the dark phase is aluminium. In addition to these two phases, a third phase with an intermediate grey colour is also visible, indicated by white arrows in the microscopic images. Both iron and aluminium are present in these areas.

**Figure 10:**
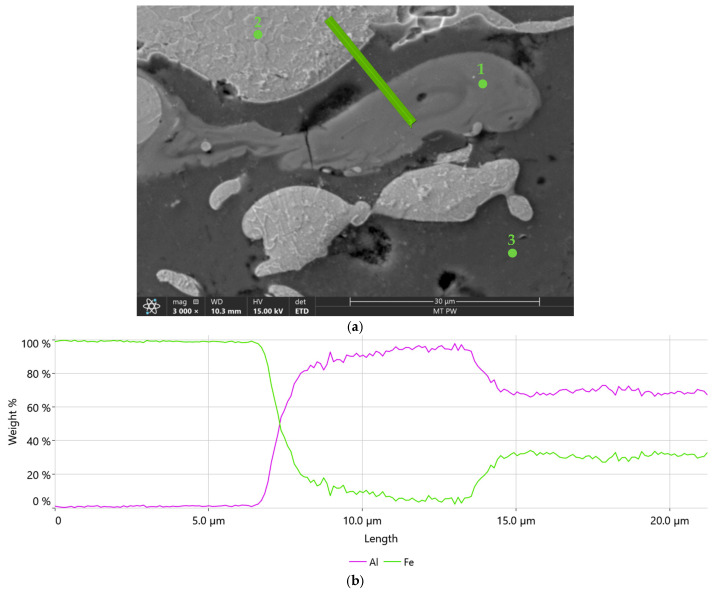
Microstructure of the in situ phase formed region (**a**) and linear distribution of Fe and Al concentration in the 1Ar coating (**b**).

Figure 10 shows an example microstructure of the 1Ar coating with a newly formed phase at a higher magnification and the distribution of aluminium and iron concentrations along the analyzed line passing through all the components of the microstructure. These studies confirm that in the matrix composed of aluminium and iron layers there is a grey phase that contains both Fe and Al. It is also clearly visible that the concentration of aluminium in this in situ formed phase is higher than that of iron. This is confirmed by the results of point analysis in three characteristic phases of the coating (Table 5). Taking into account the mutual proportions of iron and aluminium in point 1, in accordance with the Fe-Al phase equilibrium system [30], it can be concluded with high probability that in this area, the intermetallic FeAl_3_ phase was formed in situ during the spraying process. Table 6 presents the chemical compositions of the Fe-Al system phases formed in situ, in the areas marked with white arrows in Figure 7a, Figure 8a, and Figure 9a. The mutual proportions of the components indicate that the FeAl + FeAl_2_ phases (2Ar coating), FeAl_2_ phase (3Ar coating), and Fe_2_Al_5_ phase (3Air coating) were formed, respectively. According to [31], the Fe_2_Al_5_ phase forms first at high temperatures, just below the melting point of Al. This suggests that the coating sprayed in air was formed at the highest temperature. Moreover, Wang et al. [31] stated that the kinetics of FeAl_3_ phase growth is much slower than that of the FeAl and FeAl_2_ phases. This may indicate a limited volume fraction of the FeAl_3_ phase formed in situ. Hence, it can be concluded that the Fe_2_Al_5_ and FeAl_2_ phases dominate in the Fe-Al coatings. From the point of view of mechanical properties, both of these phases have high mechanical properties, as demonstrated in [32]. However, the Fe_2_Al_5_ phase has better resistance to brittle fracture and greater hardness than the FeAl_2_ phase.

**Figure 11:**
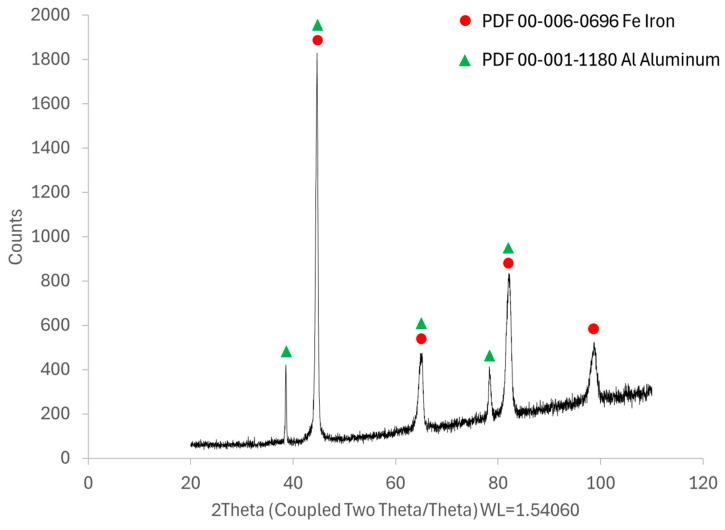
XRD patterns of 3Air coating.

Although intermetallic phases from the Fe-Al system were formed in the sprayed coatings, their share is small. The quantitative analysis of the intermetallic phases showed that for the coatings sprayed in air, the volume fraction of intermetallic phases was about 3%, while for the coatings sprayed in argon it was, on average, about 0.7%. In addition, the identified FeAl, FeAl_2_, FeAl_3_, and Fe_2_Al_5_ phases occur simultaneously in individual coatings. Therefore, the share of a specific phase in the analyzed coating is much lower than the aforementioned value. It is known that the intensity of the diffraction signal in the XRD method depends mainly on the volume fraction of the phases. In this case, the share of intermetallic phases is below the detection threshold in XRD. Due to this, even in the diffraction pattern obtained for the coating with the highest volume fraction of intermetallic phases, only peaks originating from Fe and Al were observed (Figure 11). Therefore, at this stage, the identification of intermetallic phases formed in situ is mainly based on SEM EDS studies.

The surface distributions of elements also indicate a higher concentration of oxygen in the areas where aluminum occurs and in places where pores are present (Figure 6, Figure 7, Figure 8 and Figure 9). This is undoubtedly due to the higher chemical affinity of aluminum for oxygen compared to iron. The chemical composition (EDS) results of the entire cross-sectional area of the coatings, shown in Figure 6, Figure 7, Figure 8 and Figure 9, are presented in Table 7. The obtained results clearly indicate that the share of iron and aluminum is generally at a similar level. On the other hand, a significantly increased oxygen content is clearly visible in the coatings sprayed in air.

### 3.2. Surface Roughness Analysis

**Figure 12:**
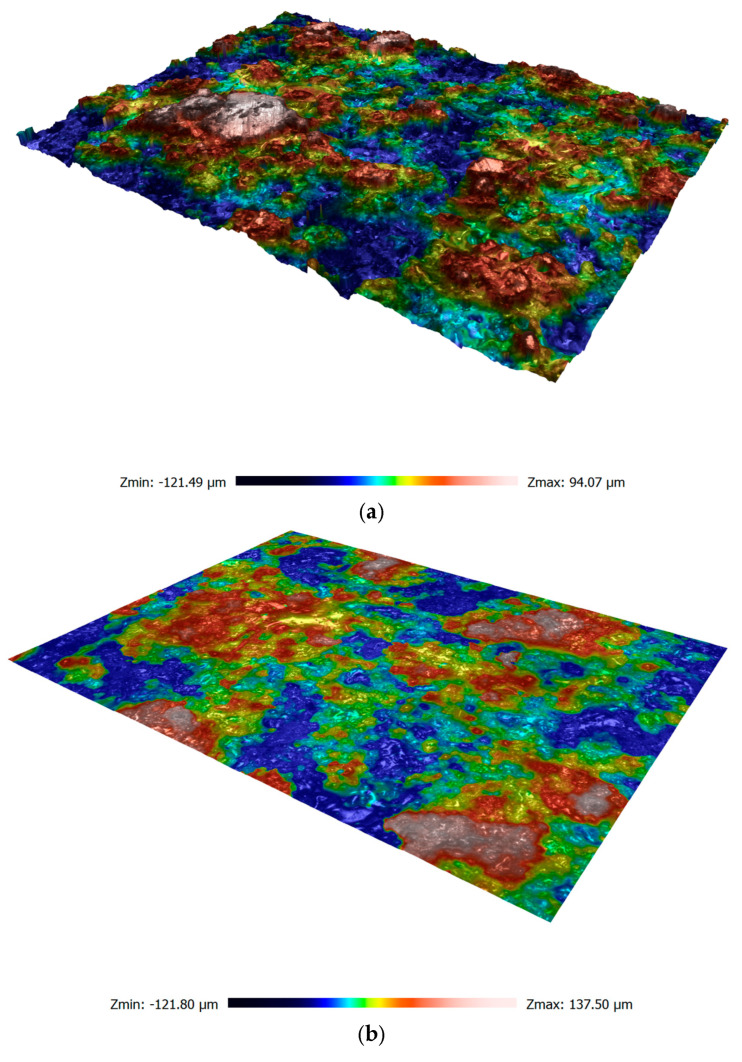

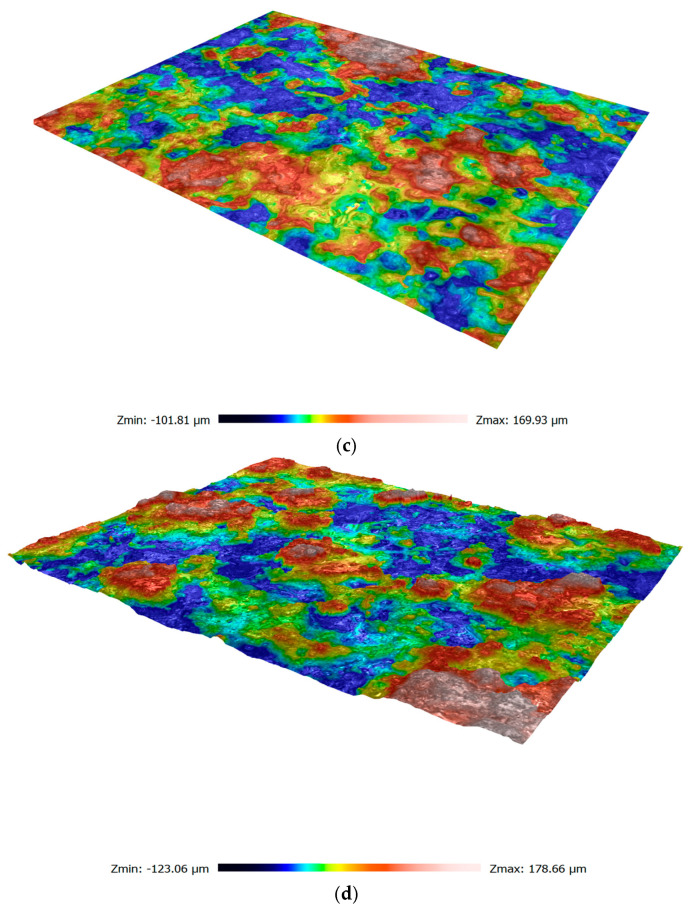
Surface roughness profile of sprayed coatings: (**a**) 1Ar, (**b**) 2Ar, (**c**) 3Ar, and (**d**) 3Air.

The roughness measurements were made on the surface of the coating. Figure 12 shows an example of the roughness profile for a sample made in argon. Table 8 presents the value of the average Sa parameter for all sample variants. Based on surface roughness measurements of samples made in argon, it was observed that as the number of spraying passes increased, the roughness parameter Sa also increased. In Figure 5a–c, particles with a nearly spherical shape are clearly visible at the coating–substrate interface. Very rapid crystallization conditions limited the flattening of the molten wire particles. The expanding argon has a lower temperature than compressed air. As a result, the molten particles of the additional material flatten to a lesser extent when falling onto the steel substrate. Hence, with the increase in the number of applied spraying passes, the surface roughness increases. In coatings sprayed in compressed air, flattened particles that fit the steel surface are visible at the coating–substrate interface (Figure 5d). Nevertheless, in the case of a sample made using compressed air, a more than 50% increase in the roughness parameter Sa was observed. This effect is related to the greater degree of oxidation of the coating (Table 7). The lack of a liquid metal shield of the additional material and the higher temperature accompanying the formation of coatings sprayed in compressed air promote a more intensive oxidation process. In this case, iron oxides, which are porous and rather loosely bound to the substrate, have a particularly unfavourable effect on the surface roughness.

### 3.3. Adhesion of Sprayed Coatings

**Figure 13:**
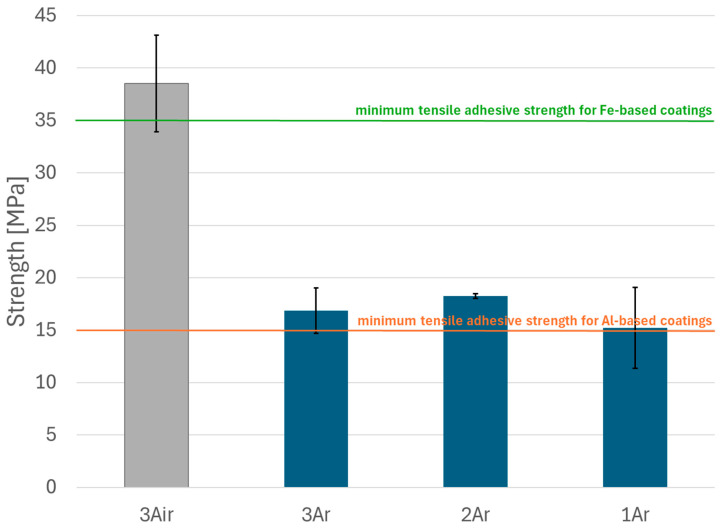
Test results of adhesion of Fe-Al coatings to the substrate.

**Figure 14:**
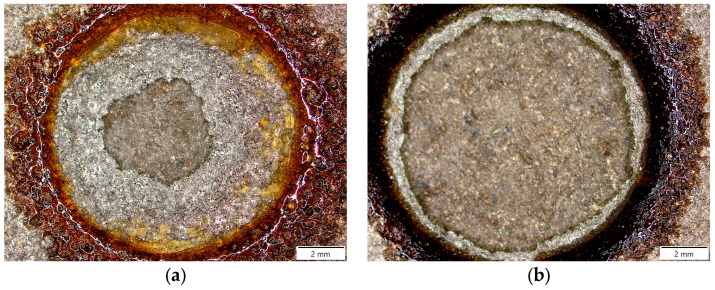

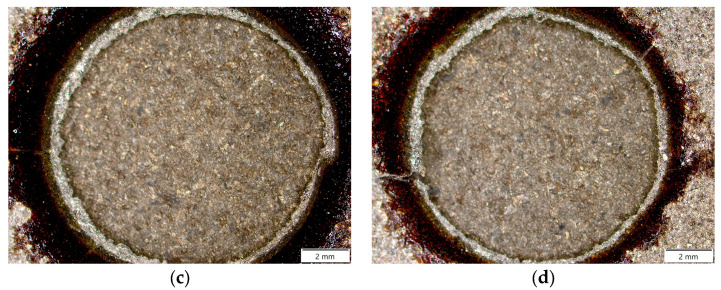
Images of the surface of selected samples after adhesion test: (**a**) 3Air, (**b**) 3Ar, (**c**) 2Ar, and (**d**) 1Ar.

The results of the adhesion of coatings to the substrate, which are the average values of three measurements, are presented in the form of a graph in Figure 13. By far the highest strength value was recorded for the variant sprayed in air. The average strength value exceeded 35 MPa, which is the minimum value required for Fe-based coatings according to [33]. Detachments for the samples thermally sprayed in air were of a mixed failure type, including coating–substrate adhesion failure, coating cohesion failure, and epoxy failure. Cohesion failure was dominant, with approximately 50% of the dolly’s surface affected after the test (Figure 14a). The use of argon as the process gas reduced the adhesive strength values, which ranged from 15.2 to 18.3 MPa. The obtained values are below 35 MPa but exceed 15 MPa, which is the minimum value required for Al-based coatings. For the samples sprayed in argon, the failure was adhesive in nature and occurred at the interface between the coating and the substrate (Figure 14b–d). The significantly higher adhesion of Fe-Al coatings to the substrate sprayed in compressed air results from the conditions under which the coatings form. The formation of Fe-Al intermetallic phases is associated with exothermic reactions [34,35,36]. On the other hand, the presence of oxygen, in addition to the formation of oxide compounds, also leads to the release of heat. The standard value of the enthalpy of formation for Al_2_O_3_ has a much higher exothermic potential than that of Fe-Al compounds [37], which in turn causes a much greater thermal activation of the substrate. The higher temperature accompanying the formation of these coatings promotes a better fit of the molten particles to the developed surface of the base material. This, in turn, increases the adhesion of Fe-Al coatings to 16Mo3 steel.

## 4. Conclusions

Based on the conducted tests and analysis of the obtained results, the following conclusions can be drawn:

- The arc spraying method enabled the correct formation of Fe-Al coatings using argon as the spray gas. The microstructure of the Fe-Al coatings is layered, with a dominant presence of two phases aluminum and Fe alloy. Additionally, during the formation of the coatings, in situ intermetallic phases were formed, identified as FeAl_3_, FeAl_2_, and Fe_2_Al_5_.
- A higher proportion of intermetallic phases from the Fe-Al system is formed in the coatings sprayed in air, approximately 3%, compared to an average of 0.7% in coatings sprayed in argon.
- The coating–substrate interface was continuous, although in the coatings sprayed with compressed air, the higher temperature during coating formation resulted in the better fitting of Fe-Al particles to the substrate.
- As a result, the adhesion of the coatings to the substrate sprayed with compressed air is significantly higher.
- Fe-Al coatings sprayed in argon had a much lower oxygen content compared to those sprayed in compressed air. Consequently, the higher degree of oxidation led to a higher roughness in the coatings sprayed in compressed air.

## Figures and Tables

**Table 1:** The chemical composition of Al (CastoMag 45203) and Fe (CastoMag45803) wires.

Material	Elements, Wt%					
	C	Si	Mn	Ti	Fe	Al
CastoMag 45203 1.6 mm	0.1	1.0	1.7	-	Bal.	-
CastoMag 45803 1.6 mm	-	5.0	-	0.1	-	Bal.

**Table 2:** The chemical composition of 16Mo3 steel [28].

Material	Elements, Wt%									
	C	Si	Mn	P	S	Cr	Ni	Mo	Cu	Fe
Steel 16Mo3	0.12–0.2	0.35	0.4–0.9	0.025	0.01	0.3	0.3	0.25–0.35	0.3	Bal.

**Table 3:** Arc spraying parameters.

Parameter	Value
Arc voltage	34 V
Current	250 A
Electrode wire diameter	1.6 mm
Wire feed	2.5 m/min
Spraying gas pressure	0.25 MPa

**Table 4:** Thicknesses of Fe-Al coatings.

Sample Designation	Thickness, µm	Porosity, %
1Ar	240	9.42 ± 0.98
2Ar	544	10.4 ± 1.23
3Ar	1180	10.11 ± 1.63
3Air	1020	12.25 ± 1.34

**Table 5:** Chemical composition of individual components of the 1Ar coating microstructure, according to Figure 10a.

Element	Weight, %		
	Point 1	Point 2	Point 3
O	1.4	0.0	2.8
Al	62.6	0.5	90.8
Si	3.8	1.1	3.0
Fe	32.1	97.1	3.3
Mn	0.0	1.4	0.0

**Table 6:** Chemical compositions of the in situ formed phases shown by white arrows in Figure 7a, Figure 8a, and Figure 9a.

Element	Weight, %		
	2Ar	3Ar	3Air
O	0.0	0.0	0.0
Al	46.7	49.5	53.7
Si	3.8	3.0	3.5
Fe	49.5	46.7	42.8
Mn	0.0	0.8	0.0

**Table 7:** Chemical composition in the cross-section of the sprayed Fe-Al coatings.

Sample Designation	Chemical Composition, Wt%					
	Al	Fe	Si	Mn	O	N
1Ar	44.5	50.5	1.8	0.8	2.3	-
2Ar	49.5	47.5	2	0.7	0.3	-
3Ar	44.8	51.4	2	0.8	1	-
3Air	31.2	61.9	1.5	0.6	4.1	0.7

**Table 8:** The average Sa parameter for all samples.

Sample Number	Sa, μm	Sq, μm
1Ar	24.206 ± 2.846	30.806 ± 3.837
2Ar	27.180 ± 2.397	37.586 ± 2.164
3Ar	29.160 ± 4.238	37.322 ± 3.020
3Air	52.412 ± 7.967	55.578 ± 10.824
0 ^1^	21.725 ± 0.520	27.756 ± 0.855

^1^ Sample number 0—substrate, blasted steel.

## Data Availability

The original contributions presented in this study are included in the article. Further inquiries can be directed to the corresponding author.
